# First principles modelling of the ion binding capacity of finger millet

**DOI:** 10.1038/s41538-024-00270-1

**Published:** 2024-05-14

**Authors:** Wei Cong Matthew Yong, Apramita Devi, Tsair-Fuh Lin, Helen F. Chappell

**Affiliations:** 1 School of Food Science and Nutrition, University of Leeds, Leeds, UK; 2 Department of Viticulture and Enology, University of California, Davis, CA USA; 3 Department of Environmental Engineering, National Cheng Kung University, Tainan, Taiwan

**Keywords:** Glycobiology, Molecular modelling

## Abstract

Finger millet, a cereal grain widely consumed in India and Africa, has gained more attention in recent years due to its high dietary fibre (arabinoxylan) and trace mineral content, and its climate resilience. The aim of this study was to understand the interactions between potassium (K^+^), calcium (Ca^2+^) and zinc (Zn^2+^) ions and the arabinoxylan structure and determine its ion-binding capacity. Three variations of a proposed model of the arabinoxylan structure were constructed and first principles Density Functional Theory calculations were carried out to determine the cation-binding capacity of the arabinoxylan complexes. Zn^2+^-arabinoxylan complexes were highly unstable and thermodynamically unfavourable in all three models. Ca^2+^ and K^+^ ions, however, form thermodynamically stable complexes, particularly involving two glucuronic acid residues as a binding pocket. Glucuronic acid residues are found to play a key role in stabilising the cation-arabinoxylan complex, and steric effects are more important to the stability than charge density. Our results highlight the most important structural features of the millet fibre regarding ion-storage capacity, and provide valuable preliminary data for confirmatory experimental studies and for the planning of clinical trials where the bioavailability of bound ions following digestion may be tested.

## Introduction

Mineral deficiencies (e.g. iron, calcium and zinc) are some of the leading nutritional problems worldwide, affecting human physical and mental development across the spectrum of high, middle and low-income countries^1,2^. While food fortification and mineral supplementation are globally accepted interventions to combat these deficiencies^3^, the lack of trace mineral bioavailability in some of these mineral-enriched foods remains a significant concern. Nutrients such as dietary fibre, polyphenols, phytic acid, and proteins, which are intrinsic nutritional components, have all been reported to bind with mineral ions, sometimes reducing the ion bioavailability^4,5^.

Millets are a ‘superfood’ at the forefront of mineral fortification initiatives in developing countries, especially in Asia and Africa, where this grain is often the major staple food for large sections of the population^6^. Indeed, the United Nations General Assembly declared 2023 to be the International Year of Millets, to promote awareness of the vital importance of this crop to millions of people. Worldwide millet production has reached ~33.6 million tonnes per year, of which India accounts for 35.3% of the total^7^. Millets are a drought-resilient crop^8^ genetically adapted to suit hot, dry climates^9^, with high levels of macronutrients (dietary fibre and protein) and minerals (iron, calcium and zinc). The most important species of commercially produced millet are pearl (*Cenchrus americanus* (L.) Morrone; *Pennisetum glaucum*), finger (*Eleusine coracan*), foxtail (*Setaria italica)*, and proso (*Panicum miliaceum*). There is, in addition, a number of other more minor millet species which are often cultivated in specific geographic areas, for example, Kodo (*Paspalum scrobiculatum* (L)), Little (*Panicum sumatrense*) and Barnyard (*Echinocloa esculenta A*.)^10^. Notably, each millet species is rich in a particular mineral; for example, pearl millet is rich in iron, finger millet in calcium and foxtail millet in zinc^6,11^.

An important concern in using millet as the solution to mineral deficiency in low and middle-income countries, through biofortification or supplementation, is the high antinutritional factors (ANFs) such as phytates, tannins, oxalates and dietary fibre (DF) content. To date, phytate is the most studied antinutritional factor studied for millets, with limited focus on other ANFs^12^. Millets are reported to be high in a mixture of carbohydrate polymers such as arabinoxylans, hemicelluloses and glucans^13–16^, and it has long been a concern that such dietary fibres play a potentially detrimental role in trace mineral bioavailability^4^. However, the chemistry of mineral-fibre complexation is still ambiguous. It remains unclear whether this effect on mineral bioavailability is valid equally for all types of dietary fibres^4,5^.

A limited amount of work has been undertaken on the structural characterisation of the arabinoxylans, glucans and celluloses in millets^14,15,17–22^. However, in cereal grains such as millets, the main type of hemicelluloses (dietary fibres) are arabinoxylans, which are predominantly found in the bran layer^23^. For example, analysis of foxtail millet found that the bran consisted of 79.22% dietary fibres, of which 47.83% was the insoluble hemicellulose, itself composed of 88.89% arabinose and xylose^23^. More specifically, for finger millet, spectroscopic studies using carbon and proton nuclear magnetic resonance (^13^C NMR, ^1^H NMR) and Fourier transform infrared spectroscopy (FTIR) revealed that the structure was made up of a β-d (1,4)-linked xylan backbone, with branching on the main chain predominantly made up of arabinose residues and uronic acid residues^18^. Similarly, Nandini and Salimath^15^ found that the uronic acid content in pearl millet was about 6%, and these uronic acid residues were substituted across the xylan backbone chain. Only one study has explicitly examined ion binding, using simple supplementation experiments on sorghum and finger millet^24^. That work indicated that nutritionally significant minerals (iron, calcium and zinc) vary in their affinity for the bulk dietary fibres, with calcium being the most likely to bind.

A small number of studies have focused on the influence of dietary fibre on the mineral bioavailability in millets using in vitro estimations only^25,26^. Amalraj and Pius^25^, examining both pearl and finger millet, found that the bioavailable percentage of calcium, determined through a synthetic-digestion process, was marginally lower in finger millet (28% raw; 28.6% cooked) than pearl millet (29.8% raw; 30.3% cooked) and wheat (34.9% raw; 34.8% cooked), even though the total calcium, determined by atomic adsorption spectroscopy, was more than seven times higher than pearl millet, and six times higher than wheat. This lower calcium bioavailability corresponds to a higher DF content of finger millet compared to other cereals.

Lestienne et al.^26^ found that fibres and tannins play an important role in reducing in vitro iron and zinc bioavailability for pearl millet by chelating the high proportion of the mineral in grain hulls. However, none of the studies evaluated the role or chemistry of specific structural components of dietary fibre on mineral binding and bioavailability. Additionally, it must be remembered that in vivo, although humans are unable to breakdown the arabinoxylan structures found in millet, gut microbiota (particularly in the colon) produce a vast array of xylanolytic enzymes capable of both degrading the xylan backbone, and cleaving the side-chain residues^27,28^. This suggests that ion-binding within the arabinoxylan structures is likely to be a positive element of this crop’s nutrient-delivering capacity and highlights the difficulty in assessing bioavailability in vitro.

Employing state-of-the-art first-principles computational modelling software, such as the CAmbridge Serial Total Energy Package (CASTEP)^29^ Density Functional Theory (DFT) code, is a particularly effective way of probing the structure and chemistry of molecular systems, providing the atomistic and molecular detail that is missed in experimental studies. DFT has been used previously to study the interaction of a variety of mono and divalent cations with other polysaccharides such as algal and bacterial alginates, chitosan, and pectin^30–34^. These studies have demonstrated that metal cations can form complexes with other polysaccharides in which the hydroxyl and carboxylic acid functional groups of the saccharide structures play a vital role in the cation binding. However, to date, no studies have demonstrated the interaction chemistry between essential trace metals and the arabinoxylan structure of millets.

For this computational study, aimed at identifying the structural elements of the arabinoxylan chains that are responsible for ion-retention, finger millet was chosen. Finger millet is generating considerable research interest with respect to eliminating calcium deficiency in the poorest regions^35^ as it contains the highest levels of calcium of all the millets (and of most other grains) at 364 mg/100 g, as well as high potassium (443 mg/100 g) and zinc (2.5 mg/100 g) levels^6^. To date, the only two studies that assessed calcium retention from finger millet in humans^36,37^ showed calcium retention levels between 19.7 and 26%. While promising, it should be noted that each study only included eight participants, resulting in a lack of statistical power^38^. However, statistical analysis^38^ of four in vitro studies^39–42^ on the bioavailability of calcium from finger millet when processed by different methods, highlighted the potential of finger millet as a bioavailable source of calcium, with an average bioavailability of 44.1 ± 14.5% in raw or unprocessed grains, and 61.4 ± 21.5% in processed millet^38^. Interestingly, not all methods of processing resulted in increased bioavailability (e.g. expansion and popping reduced the bioavailability by close to 25%), but fermentation and germination, two processes traditionally undertaken in millet-consuming areas of the world, showed increases of 38.9 and 23.2%, respectively, compared to unprocessed controls^38^. Amongst all the millets, finger millet dietary fibre structure and chemistry is experimentally the most well-elucidated^21^ and, hence, provides strong foundations for the computational models aimed at understanding these chemical interactions between key nutrient cations and the arabinoxylan structure of millet grains. Three variations of the arabinoxylan model were constructed, based on a proposed structure of the arabinoxylans found in finger millet^21^. In these models, the main linear chain comprises xylose sugars with mono or di-substituted arabinose and glucuronic acid residues. DFT was used to obtain geometrically optimised structures of cation interactions with the arabinoxylan molecules, and the thermodynamic stability of the resulting complexes was calculated. The chosen cations were potassium (K^+^), calcium (Ca^2+^) and zinc (Zn^2+^). Potassium and calcium ions were chosen due to their high concentrations in the finger millet grains^43^, while zinc was targeted due to both its high concentration amongst the millet species^6^ and its prominence in biofortification studies of millet grains^44^.

## Results

### Molecular models

A likely structure of arabinoxylans in finger millet was proposed by Subba Rao & Muralikrishna^21^ following the isolation of purified arabinoxylans from finger millet through a series of methods—methylation, ^13^C NMR, ^1^H NMR, FTIR, GLC-MS, oligosaccharide analysis, periodate oxidation and Smith degradation. They revealed that the backbone of the structure was a β-d (1-4)-xylan, with arabinose residues being both mono- and di-substituted at the C3 and C2 positions of the xylose sugar rings. Additionally, the study indicated that the majority of arabinose substitutions were mono substitutions at the C3 position, while the prominence of doubly substituted arabinose was low^21^. The presence of uronic acid residues, found to be glucuronic acid substituted at the C2 position of the xylose rings, was also detected.

**Fig. 1:**
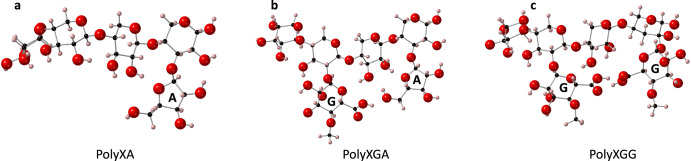
The arabinoxylan models. **a** PolyXA arabinoxylan consisting of the xylan backbone with one arabinose residue. **b** PolyXGA arabinoxylan consisting of the xylan backbone with one arabinose and one glucuronic acid residue. **c** PolyXGG arabinoxylan consisting of the xylan backbone and two glucuronic acid residues. Carbon atoms are shown in black, hydrogen in pink and oxygen in red. Residues (arabinose (A) and glucuronic acid (G)) are also labelled.

Three simplified variations of the models proposed by Subba Rao & Muralikrishna^21^ were created using *CrystalMaker*®^45^ as seen in Fig. 1a–c. Geometry optimisation using CASTEP^29^ followed, to create highly accurate representations of the arabinoxylan structures. The first model (Fig. 1a) featured the xylan backbone with an arabinose residue substituted at the C3 position of the xylose ring. The second model (Fig. 1b) featured the xylan backbone with both an arabinose residue and a glucuronic acid residue substituted at the C3 and C2 positions of the xylose rings, respectively. The final model (Fig. 1c) featured the xylan backbone with two glucuronic acid residues substituted at the C2 positions of the xylose rings. From here on, these structures will be referred to as PolyXA (Fig. 1a), PolyXGA (Fig. 1b) and PolyXGG (Fig. 1c).

All systems were optimised in a fully charge-saturated state. To this end, glucuronic carboxylic acid groups were deprotonated, as were hydroxyl groups if required. For the PolyXA system, hydroxyl ions on the arabinose and xylan backbone were deprotonated, while for the PolyXGA systems, hydroxyl hydrogen atoms were removed solely for the +2 cations (Ca^2+^, Mg^2+^). However, it should be noted that these OH groups are unlikely to be deprotonated either in the growing millet or within the gastrointestinal tract. By contrast, with a pKa of 2.93^28^, the glucuronic carboxylic acid groups are expected to be fully deprotonated under the same conditions. In this respect, the PolyXGG systems represent the most realistic structures for the doubly charged cations, although it should be noted that quantum mechanical calculations rarely return atomic charges that correspond exactly to the assumed electronic model.

### Cation interaction with the PolyXA structure

#### Binding sites

**Fig. 2:**
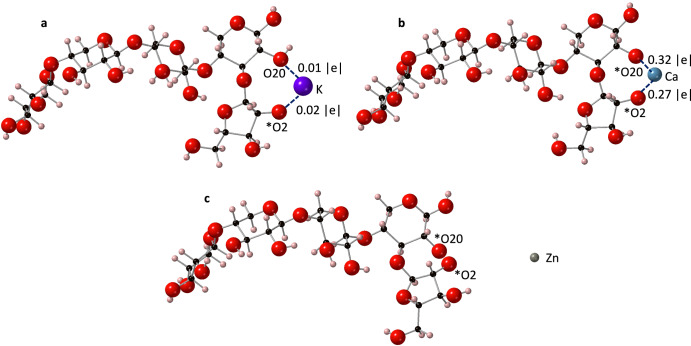
Optimised PolyXA structures with three different cations. **a** K^+^, **b** Ca^2+^ and **c** Zn^2+^. Ionic bonds to the cations are shown with dotted lines and populations are marked. Atoms with a formal charge are marked with an asterisk (*). Calcium is shown in blue, potassium in purple, zinc in grey, oxygen in red, carbon in black and hydrogen in pink.

The removal of a hydrogen atom from O2 (Fig. 2) formed an RCO^-^ group, which proved to be a stable binding position for both the K^+^ and Ca^2+^ ions following geometry optimisation (Fig. 2a, b). In the K^+^ PolyXA complex, the K^+^ ion also formed a bond with the hydroxyl group of the xylan backbone (Fig. 2a), while the Ca^2+^ ion formed a second ionic bond to the deprotonated OH group of the xylan backbone (Fig. 2b). The observations of the preferential binding positions for K^+^ and Ca^2+^ ions were coherent with other studies. Sharma et al.^46^ analysed the interaction of mono and divalent metal ions with fructose using DFT, and revealed that hydroxyl groups acted as the preferred binding sites for the metal ions. A review on calcium-carbohydrate complexes also showed the ability of calcium ions to bind to multiple oxygen atoms within a polysaccharide complex^47^. From Fig. 2c, it is clear that Zn^2+^ ions do not bind to the PolyXA structure. This result differs from previous work by Sharma et al.^46^, who revealed that zinc was capable of binding to hydroxyl groups of other sugar molecules such as α-fructose and β-fructose.

### Bond architecture

The coordination number (CN) for both K^+^ and Ca^2+^ions was found to be 2 (CN = 2), both bonds being to oxygen atoms. This is expected given that there are no other accessible oxygen atoms surrounding the potassium and calcium ions besides O20 and O2 (Fig. 2). This result aligns well with the work of Sharma et al.^46^ who showed that both potassium and calcium ions were bi-coordinated to two hydroxyl groups of α-fructose and β-fructose, in a coordination geometry that is similar to those of Fig. 2a, b.

Shannon & Prewitt^48^ provided a set of effective ionic radii that serve to study bond lengths between cations and oxygen. For the ions considered in this study, the ionic radii trend follows K^+^(1.38 Å) > Ca^2+^ (1.00 Å) > Zn^2+^(0.60 Å). Here, the average bond length of the K^+^-oxygen bond is 2.505 Å, which is greater than the average bond length of the Ca^2+^-oxygen bond (2.024 Å), following the trend in ionic radii. Tehrani et al.^49^ revealed that when potassium was involved in a bidentate bond with oxygen in sugar molecules, bond lengths ranged from 2.45 to 2.69 Å, a result that is representative of the present study.

The bond length between K^+^-O2 (2.298 Å) is shorter than the bond length between K^+^-O20 (2.711 Å), which is to be expected given the formal charge of O2 and its increased electronegativity. The bond lengths of Ca^2+^-O2 (2.045 Å) and Ca^2+^-O20 (2.003 Å) are comparable, which is also expected given that the calcium ion was bonded to the same RCO^-^ functionality.

Mulliken bond populations (Fig. 2) suggest that all cation-oxygen bonds are ionic, as defined by cation-oxygen bond populations <0.4 |e|. The weak bond populations of the K^+^-O bonds (0.01 |e| and 0.02 |e|), are considerably smaller than the average bond population of Ca^2+^-O (0.30 |e|). This can be explained by the greater charge density of Ca^2+^ compared to K^+^, resulting in considerably stronger electrostatic forces of attraction with the oxygen atom.

### Thermodynamic stability

The thermodynamic stabilities of the resulting complexes were determined by calculating the formation energy (Eq. 1, Table 1). It is evident that calcium (*E*_*f*_ = −1.06 eV) formed the most stable structures with PolyXA, followed by potassium (*E*_*f*_ = −0.19 eV). The zinc complex, however, remained thermodynamically unfavourable (*E*_*f*_ = 4.85 eV). Given that the charge density trend follows the order of Zn^2+^ > Ca^2+^ > K^+^, it is evident that the stability of the resulting cation-arabinoxylan complex is not solely due to charge density factors. Debon & Tester^50^ noted that zinc ions have no affinity for neutral polysaccharides. However, the removal of hydrogen atoms from the O2 and O20 atoms presented two RCO^−^ groups that might have been expected to bind to the positive zinc ion if charge density alone were the driver for chemical bonding. Therefore, it is necessary to consider other factors such as effective ionic radius, that can have an effect in determining the stability of the resulting cation-arabinoxylan complex. This will be discussed further in the following sections.

**Table 1:** Formation energies (eV) for the cation-PolyXA, PolyXGA and PolyXGG complexes

Structure	*E*_*f*_ (eV)		
	K^+^	Ca^2+^	Zn^2+^
PolyXA	−0.19	−1.06	4.85
PolyXGA	−1.49	−2.96	0.62
PolyXGG	−3.82	−2.99	4.82

### Cation interaction with the PolyXGA structure

#### Binding sites

**Fig. 3:**
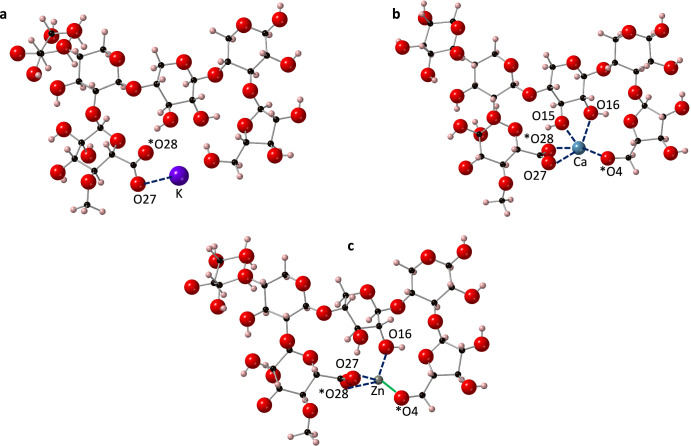
Optimised PolyXGA structures with three different cations. **a** K^+^, **b** Ca^2+^ and **c** Zn^2+^. Ionic bonds to the cations are shown with dotted lines and the one covalent bond from Zn^2+^ to O4 is shown as a solid green line. Atoms with a formal charge are marked with an asterisk (*). Note that due to resonance effects, the formal charge has become equally split between O27 and O28 upon optimisation. Bond lengths and populations are provided in Table 2. Calcium is shown in blue, potassium in purple, zinc in grey, oxygen in red, carbon in black and hydrogen in pink.

In the PolyXGA structure, the removal of hydrogen atoms from O28 and O4 created adjacent RCOO^-^ and RCO^-^ groups, which provided a stable binding pocket for both the Ca^2+^ and Zn^2+^ ions (Fig. 3b, c). In the K^+^ PolyXGA complex, the RCOO^-^ group provides the stable binding site (Fig. 3a), with the only bond to K^+^ formed in this group. It should be noted that the deprotonation of the RCOOH group of glucuronic acid results in charge resonance effects upon optimisation, such that both oxygen atoms in this group have almost equal charge. These observations corroborate the DFT work of Hills et al.^51^ who revealed that the RCOO^-^ and hydroxyl groups were favourable binding positions for mono and divalent metal ions (Na^+^, Ca^2+^, Mg^2+^) within bacterial alginate (mannuronate-guluronate) extracellular matrix complexes. Furthermore, Agulhon et al.^30^ demonstrated, also through a DFT study, that transition metals bonded to the hydroxyl or RCOO^-^ groups in metal-diuronate complexes.

### Bond architecture

Bond populations and lengths are shown in Supplementary Table 1. K^+^ was bonded to just one oxygen atom in the PolyXGA complex (CN = 1), making this the lowest CN when compared to Ca^2+^ (CN = 5) and Zn^2+^ (CN = 4). While the Zn^2+^ ion has a greater charge density than the Ca^2+^ ion, the difference in CN between the two cations can be explained by their respective sizes (ionic radii of Ca^2+^ > Zn^2+^), whereby larger cations are able to form more bonds within complexes^52^. Hills et al.^51^ also noted that alkaline earth ions were capable of forming five or six bonds with oxygen atoms of hydroxyl and carboxylate groups in other (alginate) polysaccharides, a result that was mirrored in the present study.

The bond length of the K^+^-oxygen bond was 2.634 Å, which is greater than the average bond length of the Ca^2+^-oxygen bonds (2.364 Å) and Zn^2+^-oxygen bonds (2.040 Å). The differences in bond lengths can be explained by charge density where Zn^2+^ > Ca^2+^ > K^+^, and a greater charge density would result in stronger electrostatic forces of attraction with the oxygen atom, and hence, a shorter bond length^49^. Agulhon et al.^30^ reported DFT-calculated average bond lengths of Ca^2+^ to oxygen of around 2.4 Å, which are very similar to the results presented here. Earlier, using X-ray diffraction, Bugg^53^ reported that the bond lengths of calcium to oxygen were between 2.38 to 2.54 Å in a calcium-lactose complex. Moving to the zinc-oxygen bonds, in two alternative saccharide monomer complexes (α-fructose and β-fructose), calculated using DFT, Zn^2+^-oxygen distances were found to be in the region of 2.0 Å. These previous results correspond well with the results presented here.

In the PolyXGA-calcium system, the bond length of Ca^2+^-O28 (2.384 Å) was similar to that of Ca^2+^-O27 (2.355 Å), despite O28 being the formally charged atom. This can be explained by resonance effects at the RCOO^-^ group, where the delocalisation of electrons has resulted in the negative charge being shared between the O28 and O27 atoms. The bond lengths of the Ca^2+^-RCOO^−^ bonds were, however, greater than the bond length between Ca^2+^-O4 (2.056 Å). This suggests that the interaction between Ca^2+^ and the RCO^-^ functionality of the arabinose residue was stronger than the RCOO^−^ functionality of the glucuronic acid residue. The favouring of the monodentate (RCO^-^ group) over the bidentate (RCOO^−^) bond was also seen with Zn^2+^, where the shortest bond length between Zn^2+^ and oxygen was between Zn^2+^ and O4 (1.82 Å). Once again, this suggests that the RCO^-^ functionality interacted most strongly with the cations. By contrast, Hills et al.^51^ noted that the cation and RCOO^−^ interactions in models of *Pseudomonas aeruginosa* bacterial alginate were the strongest and formed the shortest bonds compared to other oxygen groups. However, it should be noted that at physiological pH, the arabinose RCOH group would not be expected to be deprotonated. This is further explored in the PolyXGG section below.

Mulliken bond populations (Supplementary Table 1) suggest that all cation-oxygen bonds are ionic, except for the Zn^2+^-O4 bond (0.49 |e|). The ability of zinc to form covalent bonds with oxygen was also highlighted by Agulhon et al.^30^, who showed that both zinc and a number of transition metals, were able to form covalent bonds with oxygen in cation-sugar complexes. The bond population of the K^+^-O bond (0.01 |e|) is much weaker than the average bond population of both the Ca^2+^-O bonds (0.12 |e|) and the Zn^2+^-O (0.22 |e|) bonds, due to the greater charge densities of Zn^2+^ and Ca^2+^.

### Thermodynamic stability

The thermodynamic stabilities of the resulting complexes (Table 1) were determined by calculating the formation energy (Eq. 1). It is evident that Ca^2+^ ions (*E*_*f*_ = −2.96 eV) formed the most stable structures with PolyXGA followed by K^+^ (*E*_*f*_ = −1.49 eV), which is also thermodynamically stable, and Zn^2+^ (*E*_*f*_ = 0.62 eV), which is unfavourable. A study by Hills et al.^51^ also revealed that Ca^2+^, compared to other mono (Na^+^) and divalent (Mg^2+^) cations, formed the most stable complexes amongst bacterial alginates. Given that the charge density of Zn^2+^ ions is greater than both potassium and calcium ions, one would expect Zn^2+^ to have formed the most stable complexes. Therefore, it is evident that the stability of the resulting cation-arabinoxylan complex is not solely due to charge density factors. Steric factors appear to play a much greater role; due to their smaller size, zinc ions have to get closer to other atoms or groups to be within the bond-forming range, which limits the number of bonds that can be formed. Therefore, it is likely that the zinc ions have to induce greater torsional changes in the PolyXGA structure to create stable chelation sites for binding, which results in an unstable complex.

### Cation interaction with the PolyXGG structure

#### Binding sites

**Fig. 4:**
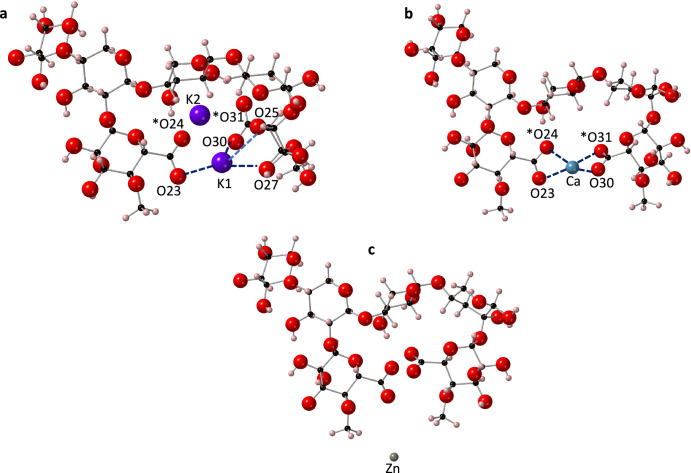
Optimised PolyXGG structures with three different cations. **a** K^+^, **b** Ca^2+^ and **c** Zn^2+^. Ionic bonds to the cations are shown with dotted lines. Atoms with a formal charge are marked with an asterisk (*). Note that due to resonance effects, upon optimisation, the formal charge has become equally split between O23 and O24, and O30 and O31. Bond lengths and populations are provided in Supplementary Table 2. Calcium is shown in blue, potassium in purple, zinc in grey, oxygen in red, carbon in black and hydrogen in pink.

The deprotonation of O24 and O31 atoms created two adjacent RCOO^-^ groups, which formed a stable binding position for both the potassium and calcium ions (Fig. 4a, b). For charge balance, two potassium ions were included in this simulation. In the potassium PolyXGG complex, the RCOO^-^ and hydroxyl groups provided a stable binding position for the K1 ion, while the K2 ion failed to bind despite being situated in a similar environment. As for calcium, the RCOO^-^ groups alone were involved in bonding. Similar observations were noted by DeLucas et al.^54^ where, using X-ray diffraction, the carboxyl and hydroxyl groups of glucuronate residues were bonded to the calcium ions. Hills et al.^51^ also demonstrated that cations were capable of forming bonds to two RCOO^-^ groups in a cross-linked alginate complex. Additionally, Saladini et al.^55^ stated that in sugar acids, the oxygen from both the carboxylic and hydroxyl functionality were involved in the binding of cations. The observations from previous studies align well with the data of the present study.

From Fig. 4c, it is clear that Zn^2+^ does not bind to the PolyXGG structure. However, Saladini et al.^55^ showed that zinc ion was capable of forming complexes with other sugar acids such as galactaric acid and d-aldonic acid.

### Bond architecture

In the K^+^ PolyXGG complex, the CN for K1 is 4, while for K2, the CN is zero, with no bonds formed (Supplementary Table 2). This lack of binding to K2 is likely to be due to repulsive forces between the two potassium ions, preventing co-localisation within the binding pocket. It should be noted, however, that the bond populations are small for the K1-oxygen bonds, and even a minor deviation from an ideal position, results in non-bonding. The CN for the Ca^2+^ complex is also 4, but the bonding varies considerably between the two structures. In the K^+^ complex, the one bonding ion forms very weak ionic bonds (0.02 |e|) to one oxygen atom (O23, O30) of each of the deprotonated RCOOH groups on the adjacent glucuronic acid residues. Notably, although it was O24 and O31 that were the formally deprotonated oxygen atoms, following optimisation, and through the establishment of resonance effects, it is O23 and O30 that have the greater negative charge, −0.73 e (O23) compared to −0.67 e (O24), and −0.74 e (O30) compared to −0.69 e (O31). In addition, there are two slightly stronger hydrogen bonds from K1 to a ring oxygen (O25) and a hydroxyl oxygen (O27). Similarly, Tian et al.^56^ noted that, using FTIR, only one oxygen atom from each RCOO^-^ group was involved in the bonding of K^+^ ions in a K^+^-galactarate complex. It is also evident that in this complex, the cation-RCOO^−^ bonds are now shorter and stronger (in contrast to the PolyXGA system), which corresponds more closely to the results of Hills et al.^51^ who made the same observation in binding pockets composed of at least two carboxylic acid residues. In contrast to the K^+^ ions, the Ca^2+^ ion sits squarely in the binding pocket between the two adjacent RCOO^-^ groups, bonding to all four oxygen atoms.

**Fig. 5:**
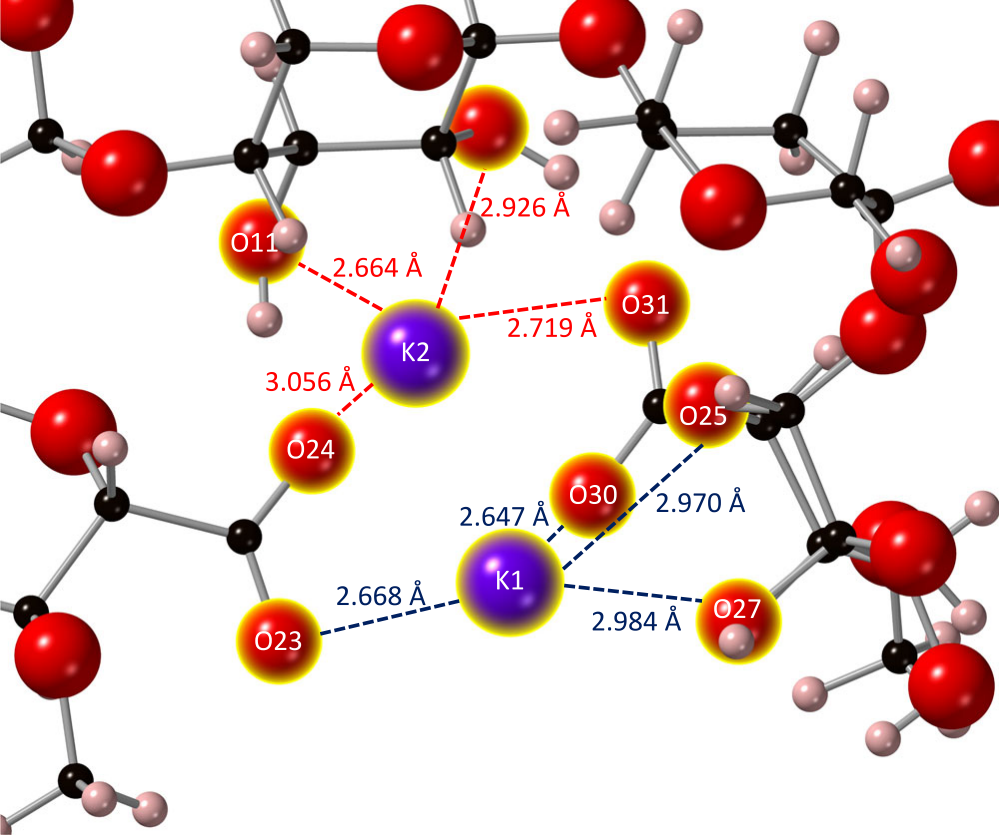
A close-up view of the two K^+^ ions in the PolyXGG complex, illustrating the similar binding pocket environments. The Mulliken-predicted bonds to K1 are shown as blue dotted line, and bond distances (not predicted as bonding) to K2 are shown with red dotted lines.

The bond lengths between the calcium ion and oxygen atoms were all within 0.12 Å of each other. This suggests that the interactions between Ca^2+^ ions and the oxygen atoms of the carboxylic functionality of the glucuronic acid residues were similar in strength. This result can once again be explained by the stabilisation of the RCOO^-^ functionality due to positive steric effects with respect to the size of both the binding pocket and the Ca^2+^ ion. With regards to the K^+^ ions, it is interesting to note that although no bonding is predicted to K2 (using Mulliken population analysis), it is nevertheless residing in the vicinity of the second RCOO^−^ group, as illustrated in Fig. 5. It seems likely that the energetics of this position are very similar to that of K1, and the formation energy for the complex overall (−3.82 eV) does indeed suggest favourable binding positions for both ions. It should also be noted that with the two glucuronic acid residues, the K^+^ now forms its strongest (and shortest) bonds (see Supplementary Table 2).

Mulliken bond populations suggest that all cation-oxygen bonds are ionic. The average bond population of K^+^-O is 0.04 |e|, which is significantly smaller than the average bond population of the Ca^2+^-O bonds (0.08 |e|). This is expected given that the charge density of Ca^2+^ is greater than K^+^, resulting in stronger electrostatic forces of attraction with calcium ions. However, in bacterial alginates (mannuronate/guluronate polysaccharides) average Ca^2+^-oxygen bond populations as high as 0.14 |e| have been observed^51^. These higher bond populations were found in binding sites that encompassed not just carboxylic acid groups, but a wider range of oxygen functionality, including hydroxyl and ring oxygen atoms^51^.

### Thermodynamic stability

From Table 1, it is clear that both potassium (−3.82 eV) and calcium (−2.99 eV) formed thermodynamically stable structures with the PolyXGG motif. This is in contrast to zinc (4.82 eV), which proved highly unstable. Given that the charge density trend follows Zn^2+^ > Ca^2+^ > K^+^, it might have been expected that zinc would form the most stable complex within the PolyXGG complex. Indeed, Gianguzza et al.^57^ indicated that in glucuronate complexes, Zn^2+^ formed the most stable complex compared to Ca^2+^ and Na^+^. Equally, based on charge density arguments alone, Ca^2+^ ions should have formed more stable complexes with the arabinoxylan structure than potassium ions. However, the lack of stability in zinc binding can be explained using steric factors. Due to its smaller size, Zn2+ ions require a greater torsional change of the PolyXGG backbone structure to create favourable binding sites. K^+^ and Ca^2+^ clearly have better steric compatibility with the glucuronate-glucuronate binding pockets likely to be found within finger millet.

## Discussion

The interactions between K^+^, Ca^2+^ and Zn^2+^ ions with the xylan backbone (four xylose sugar rings) of the arabinoxylan structure were also calculated (Supplementary Tables 3, 4 and Supplementary Fig. 1). Once again, the results showed that Zn^2+^ was thermodynamically unfavourable, while both Ca^2+^ and K^+^ ionically bonded to the RCO^−^ and hydroxyl groups of the backbone. Ca^2+^ formed the most stable complex (−0.99 eV), followed by K^+^ (−0.42 eV). Initially, the addition of the arabinose residue (PolyXA) improved the stability of the Ca^2+^-arabinoxylan complex, but this was not the case for Zn^2+^. However, for both Ca^2+^ and K^+^, the addition of the glucuronic acid residue to the xylan backbone improved the thermodynamic stability of the cation-arabinoxylan complex considerably, as seen in the PolyXGA and PolyXGG structures. The PolyXGG structure, which corresponds most closely to that likely to be found within millet, was able to form the most stable complexes of all with Ca^2+^ and K^+^.

The inability of Zn^2+^ to form stable complexes with any of the PolyX structures can be explained by its smaller size, which prevented it from finding a stable multi-oxygen binding pocket across any of the structures. A previous study by Reese et al.^58^ looked at the thermodynamic stability of both Eu^3+^ and Cm^3+^ with glucuronic acid complexes and found that despite having a smaller charge density, the larger Cm^3+^cation was able to form more stable structures than the smaller Eu^3+^ cation. Furthermore, the addition of Eu^3+^ caused a distortion of the glucuronic acid structure, reducing the overall thermodynamic stability of the complex. This phenomenon corresponds well with the results obtained for Zn^2+^, which, despite having a higher charge density, showed an inability to bind to the PolyXA and PolyXGG structures. Although Zn^2+^ was able to bind to the PolyXGA structure, the resulting complex remained thermodynamically unfavourable, suggesting instability of the resulting complex. Hence, it is evident that the ionic radii of the cations play a crucial role in determining the overall stability of cation-sugar complexes where the xylan backbone appears relatively inflexible.

In the present study, Ca^2+^ formed the most stable complexes in the PolyXA and PolyXGA complexes, while the formation energy of K^+^ suggested it formed the most stable PolyXGG complex. Zinc ions were incapable of forming any thermodynamically favourable structures with the arabinoxylan molecule, which suggests that Zn^2+^ would not bond to this fraction of the finger millet grains. Indeed, these data help to explain the documented nutrient levels of zinc (2.5 mg/100 g), potassium (443 mg/100 g) and calcium (364 mg/100 g) in finger millet, where zinc is present at much lower concentrations^6^. Given the weak binding of zinc in our models, it is unsurprising that measured values are low in fresh, raw grains.

However, free phenolic acids are also present in finger millets^20^, and these compounds could potentially form favourable interactions with zinc and other ions, although to date, no published data have been reported on this. Given these results, it might be suggested that zinc may be relevant as a supplemental ion only (where zinc compounds are simply mixed with millet products, e.g. flours). Given that the formation energies for all calcium and potassium PolyX complexes were thermodynamically favourable, both calcium and potassium ions are likely to exist as bound minerals within the arabinoxylan complex. The bioavailability of the minerals bound to dietary fibre is still a point of discussion, considering factors such as structure, solubility and fermentability of fibre in the gut^5^. However, in vitro studies examining cooking and processing methods on calcium bioavailability, suggest millet has a value of around 28%^38^, and the two previous human studies on retention suggest around 25% of calcium is retained within the body^36,37^. Although these bioavailability and retention figures are not particularly large, finger millet has an extremely high level of calcium, which means that overall finger millet is a particularly good source of this mineral. Although humans do not produce xylanolytic enzymes, it is well established that gut microbiota produces a vast array of such molecules, which are capable of degrading the xylan backbone and cleaving the side-chain residues. This suggests that tight ion-binding, as observed here with potassium and calcium, does not necessarily mean that bioavailability is particularly low, as discussed above. Indeed, it is likely that these ions will be released within the gastrointestinal tract to some extent, as the microbiota-produced degradative enzymes breakdown the structures and disrupt, or completely collapse, the ion-binding pockets we have identified. Earlier studies have reported that the more fermentable and less viscous the bound fibre, the greater the tendency for mineral release^59^. Currently, it is known that arabinoxylans *are* metabolised in the colon to yield short-chain fatty acids (SCFAs), which eventually would lower the gut luminal pH, thus aiding in mineral absorption^60^. Furthermore, the fermentation characteristics of arabinoxylans depend strongly on their structural properties. Complex branching patterns in arabinoxylans from wheat and corn have been shown to decrease fermentation rates in vitro in the presence of human faecal bacteria^61^, and the degree of fermentation appears to be lower with fibres that contain a large arabinose component or a larger arabinose:xylose ratio^62^. Hence, the bioavailability of bound minerals may greatly depend on molecular weight, degree of substitution, branching pattern and phenolic acid substitution on the arabinoxylan^28^. Therefore, future experimental validation using in vivo models and human subjects is important to understand the implication of structural variations of millet fibres on the binding of minerals and its bioavailability, as is the structural and chemical characterisation of the different arabinoxylan molecules found across the main varieties of millet.

For both the calcium and potassium ions, we revealed that the addition of glucuronic acid residues improved the thermodynamic stability of the PolyX-cation structures, and the cation-PolyXGG complex was the most stable structure of all compared. Our results suggest that even though glucuronic acid residues are present in smaller concentrations than arabinose residues^20^, they play a vital role in determining the overall stability of the cation-arabinoxylan complex, and will perhaps be a limiting factor in terms of cation-binding concentrations. In this study, we utilised the proposed arabinoxylan structure in finger millet by Subba Rao & Muralikrishna^21^, which encompasses predominantly arabinose residues over glucuronic acid residues, across the xylan backbone. Given the results presented here, it would therefore be interesting to determine strains of finger millet that have greater concentrations of glucuronic acid residues within the arabinoxylan structure for targeted breeding programmes, but also to assess the in vivo digestibility of these branched structures. Puranik et al.^35^ highlight the current interest in finger millet for just such biofortification studies, but they note a frustrating lack of progress in this area. This has been due to many factors, including the difficulty in hybridising a self-fertile crop (as finger millet is), and a lack of robust donor germplasms that are known to have high-calcium stability in multiple different environments.

While DFT is a highly accurate method of computing geometrically optimised structures, the limitation of this model is the use of a small, single-chain structure of the arabinoxylan molecule. Within the living millet structure, it is possible that multiple overlapping arabinoxylan structures would exist, which could present more stable chelation sites and display the cross-linking stability of arabinoxylan structures with cations. Furthermore, interactions of other trace elements (e.g. phosphorus, iron and magnesium) with the arabinoxylan structure have yet to be identified. Also, it is important to understand the interaction of dietary fibres with phytate, phenolics and tannins, which may also eventually impact the mineral bio-accessibility in the grain. Nonetheless, the results in the present study serve as an important starting point in understanding the atomic level chemistry in the millet structure, demonstrating that calcium and potassium cations are indeed capable of forming complexes with the arabinoxylan molecule, stabilised by oxygen atoms of the RCOO^-^, RCO^-^ and hydroxyl functionality of the xylan backbone and side-chain residues.

## Methods

### Computational details

All geometry optimisations were carried out using the plane-wave density functional theory (DFT) code, CASTEP^29^. A convergence-tested box of 35 Å × 20 Å × 15 Å was used for all cation-arabinoxylan complexes, and a convergence-tested cut-off energy of 900 eV was employed. A Monkhorst-Pack **k**-point grid of 1 × 1 × 1 was used to sample the Brillouin zone^63^. Ultrasoft pseudopotentials^64^ were used throughout, along with the generalised gradient approximation of Perdew–Burke–Ernzerhof for the exchange-correlation functional^65^. The energy, force and displacement tolerances for the geometry optimisation calculations were set to 1 × 10^−5^ eV atom^−1^, 0.001 Å and 0.03 eV Å^−1^, respectively. All molecules were created and visualised using *CrystalMaker*®^45^. Mulliken^66^ bond populations were calculated to determine the nature of bonds in each of the optimised ion-complexation structures with a bond population of <0.40 |e| identified as an ionic bond, and a population >0.40 |e| as a covalent bond. Chemical potentials for calcium, potassium and zinc were calculated based on their pure-metal crystalline structures in the lowest energy state configurations, namely face-centred cubic (FCC) for calcium, body-centred cubic (BCC) for potassium, and hexagonal (HCP) for zinc, as previously described^67^. The chemical potential for hydrogen was calculated from a single molecule. All chemical potential calculations were carried out at the same convergence-tested cut-off energy of 900 eV.

Formation energies were calculated using:

1 $${E}_{f}={E}_{{{\mathrm{final}}}}{{\mbox{-}}}({E}_{{{\mathrm{initial}}}}+{\mu A}^{x}{{\mbox{-}}}y\mu H)$$

where *E*_final_ represents the energy of the optimised system, *E*_initial_ is the energy of the initial arabinoxylan structure, *μ*_*A*_^*x*^ is the chemical potential of cation A with charge x and *μ*_*H*_ is the chemical potential of a single hydrogen atom, with *y* representing the number of hydrogen atoms required to balance charge x.

### Reporting summary

Further information on research design is available in the Nature Research Reporting Summary linked to this article.

### Supplementary information

Supplementary Material

Reporting summary

## Data Availability

Supplementary Information is available for this publication, which contains the most important data. Bond length and bond population data for PolyXGA and PolyXGG structures and, backbone-cation interaction data and figures are provided. The simulation input and output files required to reproduce all these findings are available to download from: Helen Chappell, Apramita Devi, Wei Cong Matthew Yong, Tsair-Fuh Lin (2023): First Principles Modelling of the Ion-Binding Capacity of Finger Millet. [Dataset]. 10.5518/1358.
