# Supplementary material for: First principles modelling of the ion binding capacity of finger millet
Source: NPJ Sci Food. 2024 May 14;8:28. doi: 10.1038/s41538-024-00270-1 (PMC11094100; doi:10.1038/s41538-024-00270-1)
Supplement: Supplementary file 1 — Supplementary Material [file 41538_2024_270_MOESM1_ESM.pdf]

# Supplementary Information

## First principles modelling of the ion binding capacity of finger millet

### *Bond length and population data for PolyXGA and PolyXGG*

**Supplementary table 1.** Bond lengths and populations found in the PolyXGA-cation complexes. Oxygen atoms marked with an asterisk belong to the $RCOO^-$ functionality.

| Structure | Bond | Bond Length (Å) | Bond Population (\|*e*\|) |
|---|---|---|---|
| PolyXGA - Potassium | K-O27* | 2.634 | 0.01 |
| PolyXGA - Calcium | Ca-O4 | 2.056 | 0.33 |
| | Ca-O15 | 2.561 | 0.06 |
| | Ca-O16 | 2.464 | 0.07 |
| | Ca-O27* | 2.355 | 0.06 |
| | Ca-O28* | 2.384 | 0.06 |
| PolyXGA - Zinc | Zn-O4 | 1.823 | 0.49 |
| | Zn-O16 | 2.122 | 0.14 |
| | Zn-O27* | 2.114 | 0.12 |
| | Zn-O28* | 2.096 | 0.13 |

**Supplementary table 2.** Bond lengths and populations found in the PolyXGG-cation complexes. K2 and Zn form no bonds with any other atoms. Oxygen atoms marked with an asterisk belong to the $RCOO^-$ functionality.

| **Structure** | **Bond** | **Bond Length (*Å*)** | **Bond Population (\|*e*\|)** |
|---|---|---|---|
| PolyXGG - Potassium | K1-O23* | 2.668 | 0.02 |
| | K1-O25 | 2.970 | 0.07 |
| | K1-O27 | 2.984 | 0.03 |
| | K1-O30* | 2.647 | 0.02 |
| PolyXGG - Calcium | Ca-O30* | 2.240 | 0.09 |
| | Ca-O24* | 2.253 | 0.08 |
| | Ca-O31* | 2.268 | 0.08 |
| | Ca-O23* | 2.362 | 0.07 |
| PolyXGG - Zinc | - | - | - |

## *Xylan backbone – cation interactions*

**Supplementary table 3.** Formation energies (eV) for the cation-Xylan backbone complexes.

| **Structure** | $\boldsymbol{E_f}$ **(ev)** | | |
|---|---|---|---|
| | $K^+$ | $Ca^{2+}$ | $Zn^{2+}$ |
| Xylan Backbone | -0.42 | -1.00 | 4.78 |

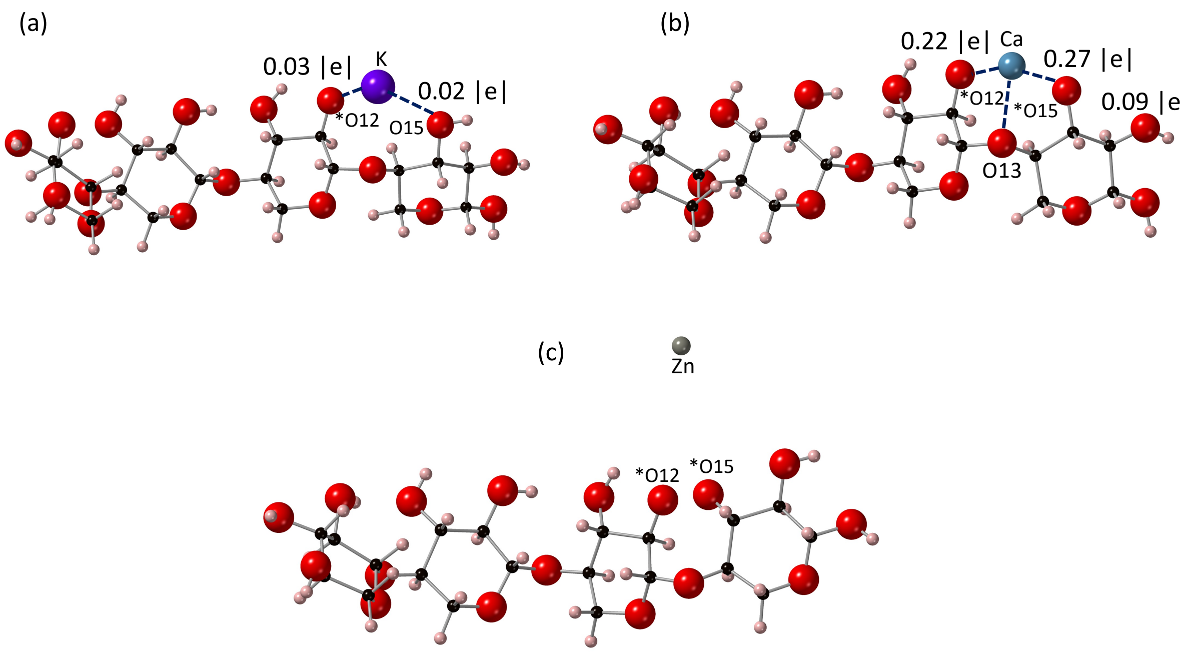


**Supplementary Figure 1.** Charge-balanced xylan backbone structures with (a) potassium, (b) calcium and (c) zinc. Ionic bonds to the cations are shown with dotted lines. Oxygen atoms with a formal charge are marked with an asterisk (*). Bond lengths and populations are provided in Table S4. Calcium is shown in blue, potassium in purple, zinc in grey, oxygen in red, carbon in black and hydrogen in pink.

**Supplementary table 4.** Bond lengths and populations found in the PolyXGA-cation complexes.

| **Structure** | **Bond** | **Bond Length (*Å*)** | **Bond Population (\|*e*\|)** |
|---|---|---|---|
| Backbone - Potassium | K-O12 | 2.367 | 0.03 |
| | K-O15 | 2.672 | 0.02 |
| Backbone - Calcium | Ca-O12 | 2.104 | 0.22 |
| | Ca-O13 | 2.564 | 0.09 |
| | Ca-O15 | 2.041 | 0.27 |
| Backbone - Zinc | N/A | N/A | N/A |
